# The Prevalence of Pseudoscientific Ideas and Neuromyths Among Sports Coaches

**DOI:** 10.3389/fpsyg.2018.00641

**Published:** 2018-05-02

**Authors:** Richard P. Bailey, Daniel J. Madigan, Ed Cope, Adam R. Nicholls

**Affiliations:** ^1^International Council of Sport Science and Physical Education, Berlin, Germany; ^2^School of Sport, York St. John University, York, United Kingdom; ^3^School of Life Sciences, University of Hull, Kingston upon Hull, United Kingdom

**Keywords:** learning styles, neuro-linguistic programming, guided discovery, brain Gym, Myers-Briggs Type Inventory (MBTI), growth mindset, action types approach

## Abstract

There has been an exponential growth in research examining the neurological basis of human cognition and learning. Little is known, however, about the extent to which sports coaches are aware of these advances. Consequently, the aim of the present study was to examine the prevalence of pseudoscientific ideas among British and Irish sports coaches. In total, 545 coaches from the United Kingdom and Ireland completed a measure that included questions about how evidence-based theories of the brain might enhance coaching and learning, how they were exposed to these different theories, and their awareness of neuromyths. Results revealed that the coaches believed that an enhanced understanding of the brain helped with their planning and delivery of sports sessions. Goal-setting was the most frequently used strategy. Interestingly, 41.6% of the coaches agreed with statements that promoted neuromyths. The most prevalent neuromyth was “individuals learn better when they receive information in their preferred learning style (e.g., auditory, visual, or kinesthetic),” which 62% of coaches believed. It is apparent that a relatively large percentage of coaches base aspects of their coaching practice on neuromyths and other pseudoscientific ideas. Strategies for addressing this situation are briefly discussed and include changing the content of coach education programs.

## Introduction

Recent years have seen the development of new research methods that have led to remarkable progress in the understanding of the neurological basis of human cognition and learning. For example, in 2011, fewer than 750 published scientific articles used findings from functional magnetic resonance imaging (fMRI) on the human brain. By the beginning of 2017, there were 32,500 fMRI studies reported in the PubMed database. fMRI and other imaging techniques enable researchers to look inside the living brain, create images that locate regions of activity associated with specific cognitive tasks, as well as reveal structural differences among individual brains (Passingham and Rowe, 2015). Our understanding of the biochemistry of the brain, intra-cellular recording, pharmacological interventions, and other technologies have developed at an accelerated pace (Pokorski, 2015). Combined with psychological research, these studies have greatly improved our understanding of the basic processes that underlie capabilities such as attention, memory, and social interaction (Mareschal et al., 2014; Immordino-Yang, 2016). Understandably, these advances have sparked a great deal of interest in the possibility of improving applied fields such as education by using this body of research (OECD, 2007; Serpati and Loughan, 2012). Consequently, there has been an accelerated growth in studies from neuroscientists, cognitive psychologists, and researchers in associated fields that have sought to apply developing insights about the brain, although many judge the current state and status of this work to be in its infancy (Bruer, 2016; Meeusen et al., 2017). At the same, a new industry has emerged that mimics many of the superficial aspects of genuine neuroscience, such as frequent use of the prefixes “neuro” and “psycho,” but that often fail to adhere to the basic tenets of scientific practice, including fair testing, peer review, and accommodating existing findings (Pigliucci and Boudry, 2013; Bailey, 2017). To date, there has not been any research into the infiltration of such pseudoscientific ideas and practices in the field of sports coaching, although anecdotal evidence suggests that they are ubiquitous.

### Pseudoscientific ideas in professional and applied contexts

Historically, the relationship between practical contexts and empirical science has been an uncomfortable one (e.g., Carnine, 2000; Fulford, 2008). There is a broad consensus within many practical contexts (e.g., coaching, sports psychology, education) that these areas should be informed by evidence, but there appears to be much less agreement about what this means in practice. As in other areas of applied science, there exists a perennial risk of the intrusion of dubious claims and practices (Lilienfeld et al., 2012; Pennycook et al., 2015), which may limit the effectiveness of applied practice and increase the risk of harm to those who experience them. This risk is particularly evident when those claims are couched in the language of neuroscience (Weisberg et al., 2008). Labels like “bad science” (Goldacre, 2009), “voodoo science” (Park, 2002), and most commonly “pseudoscience” (Lilienfeld et al., 2003) are typically used to refer to ideas or practices that seek to resemble real science, but which fail to follow its guiding principles. Many writers have sought to demarcate “real” science from pseudoscience in a wide range of fields, including clinical psychology (Tavris, 2014), social work (Thyer and Pignotti, 2016), and health care (Singh and Ernst, 2008). Yet, despite concerns regarding the proliferation of pseudoscientific ideas and practices, not much is known about their prevalence among professionals who draw upon ideas related to learning in their work (Dekker et al., 2012), among which sports coaches could be included (Jones, 2006).

The most substantial work in this area has been in school education, where studies in different countries have shown wide scale acceptance of questionable ideas and practices among teachers (e.g., Dekker et al., 2012; Gleichgerrcht et al., 2015; Pei et al., 2015). These ideas range from “neuromyths” (OECD, 2007) that have entered popular discourse, such as the idea that people only use 10% of their brains and that there are distinct “left-brain” and “right-brain” thinkers (Della Sala, 1999). Furthermore, so-called “brain-based” educational programs based on misapplied neuro-scientific research (Hyatt, 2007), and discredited theories of learning and cognitive functioning (Willingham, 2009), are often presented as the outcomes of cutting-edge neuroscientific research (Bailey, 2017).

This current paper reports on the first study of the prevalence of pseudoscientific ideas regarding the brain and learning among sports coaches. Coaches form an interesting group to consider in this regard for several reasons. First, sport coaching is a “young” academic discipline, and it draws liberally from cognate disciplines, such as psychology, teacher education, and the sport sciences (Gilbert and Trudel, 2004). It is interesting to discover whether some of the questionable ideas appearing in these more established fields also appear in an emerging field like sports coaching. Second, there have been numerous calls for sports coaching to achieve the status of a profession, with the concomitant expectation of the establishment of a defensible body of knowledge (Stodter and Cushion, 2017), so it is important to understand the current forms of knowledge and understanding. Third, the calls for professionalization have led to demands for “coach education” programs that are rigorous and evidence-based (Piggott, 2015; Stodter and Cushion, 2016), suggesting a particular focus on this area. Finally, pressures of competitive success mean that many coaches and their organizations are continually searching for new, advantageous ideas to improve their players' performances, potentially increasing their vulnerability to pseudoscientific ideas (Collins and Bailey, 2013).

### Distinguishing scientific and pseudoscientific ideas—five examples

A potential challenge facing any discussion or measurement of scientific and pseudoscientific theories is that the line of demarcation is often difficult to draw (Bailey, 2017). The classic attempt at drawing such as line was by the philosopher, Popper (1934), but his formal theory of falsifications has generally been rejected by philosophers of science for being too inclusive of non-scientific ideas, and unable to defend against *ad hoc* criticisms (Monton, 2013; Bailey, 2017). Most commentators, however, continue to endorse its central tenets broadly, such as the central importance of a critical approach, well-designed tests and a suspicion of an over-reliance on confirming evidence (Lilienfeld, 2012). Moreover, there is a much greater degree of agreement about what pseudoscience looks like (e.g., Lilienfeld et al., 2003; Koertge, 2013; Bailey, 2017), including:

- Unfalsifiability
- Absence of self-correction
- Overuse of *ad hoc* immunizing tactics designed to protect theories from refutation
- Absence of connectivity with other domains of knowledge
- Use of unnecessarily unclear language
- Over-reliance on anecdotes and testimonials at the expense of systematic evidence
- Evasion of genuine peer review
- Emphasis on confirmation rather than refutation.

### Learning styles

By far the most researched neuromyth is learning styles, and academic interest into this subject reflects its very widespread acceptance in many countries (cf. Dekker et al., 2012). In fact, the term learning styles embraces a varied set of claims, inventories, and models for assessment (Coffield et al., 2004), but the most common form of the theory promotes the “VAK” model in which some people learn best by observing (“visual learners”), some by listening (“auditory learners”), and some by doing and moving (“kinesthetic learners”). A review of studies from the UK, the Netherlands, Turkey, Greece, and China found that more than 90% of teachers agreed that students learn better when they receive information tailored to their preferred learning styles (Howard-Jones, 2014). Despite its popularity, however, there is no compelling evidence that matching formal instruction to individual perceptual strengths and weaknesses is any more effective than instruction, which is not multi-sensory specific (Rohrer and Pashler, 2012).

### Neurolinguistic programming (NLP)

NLP is a popular brain-based approach. As the name suggest, NLP seeks to align itself to the neurosciences. Its claims that eye movements give insight into thought processes, that certain language patterns can influence others' behavior, and that the skills of experts can be learned with relative ease by identifying and coding their unconscious thought processes, have bled into sport psychology, teacher education, professional development, talent identification, and other areas (e.g., Lazarus and Cohen, 2009). The scientific status of NLP is controversial, and this is largely due to a disjunction between the often-ambitious claims made on its behalf by advocates and the relative lack of serious research in support of those claims (Norcross et al., 2006; Witkowski, 2010). Carey et al. (2010) published what the authors call a “systematic review” that was strongly supportive of NLP claims. However, the authors failed to adhere to even the most basic protocols for these reviews, such as explicit inclusion/exclusion criteria and search strings, the use of multiple databases, and independent validation. Also, it included non-peer reviewed papers and essays, but contained little reference to the critical scientific literature.

### Brain-based approaches

Similarly, loose interpretations of the scientific method have been observed in numerous so-called “brain-based” approaches (Bailey, 2017). For example, the widely used Brain Gym prescribes simple movements designed to improve the integration of specific brain functions with body movements (Dennison and Dennison, 1994). Lying behind Brain Gym's activities are three main theoretical hypotheses that have been adapted from older theories: neurological re-patterning, cerebral dominance, and perceptual–motor training. None of these foundational principles, at least as they are interpreted in Brain Gym, have empirical support (Hyatt, 2007; Bailey, 2017). Scrutiny of advocacy literature gives rise to some concerns. The most apparent is that very little of this literature is published in peer-reviewed academic journals and appears in the in-house “Brain Gym® Journal,” which includes articles with quite fundamental errors in methodology, such as misinterpreting statistical significance, inappropriate control groups, and failing to account for maturational effects (Bailey, 2017).

### Myers-briggs type inventory (MBTI)

MBTI (Myers and McCaulley, 1985) is a personality measure that is based on the theory of psychological types of the psychoanalyst Carl Jung (Barbuto, 1997). It was originally developed in the 1940s as a means for analyzing characters in literature, but was later adapted to be a test for personnel selection. It is now widely used in educational and business settings. For example, the UK's Universities and Colleges Admissions Service, which manages the application process for British higher education, invites potential applicants to “Find out what you're like and what you could do,” using a short assessment evidently based on a short-form of MBTI (https://www.ucas.com/ucas/16-18-choices/find-career-ideas/buzz-quiz; accessed 09/28/17). The basic assumption of MBTI is that different vocations favor different personality orientations, and that Jung's theory provides the theoretical structure to link personality and job performance. It is generally regarded as a controversial approach, and is not widely endorsed by academic researchers in the field (Pittenger, 1993; Christiansen and Tett, 2013). Several concerns have been raised about its use. A serious worry is its reliance on Jung's typology of personality, which has long been discarded by psychological science (Domino and Domino, 2006). The ontological basis of the “types” used in the test is questionable, as is its reliance on sets of binary distinctions (such as “introverts”/“extroverts”). Jung himself thought that these dualistic definitions were mistaken (or, at least, fictions; Jung, 1921), but they were still used within the MBTI. There is a large and conflicting body of research that examines the validity of the test. Conventional psychometric analysis has often produced negative or ambivalent results (e.g., Pittenger, 1993; Bess and Harvey, 2002), and critics have raised doubts about the instrument's reliability and validity. Some studies have called the instrument's test-retest validity into question (Pittenger, 2005), and highlighted the absence of built-in scale to determine inconsistency or exaggeration in responses, making it difficult to judge when an individual is answering truthfully (Bess and Harvey, 2002). Nevertheless, the MBTI continues to be widely used in a variety of professional and nonprofessional contexts (e.g., Pittenger, 2005; Rushton et al., 2007).

### Action type approach (ATA)

Finally, the ATA is included in the list of questionable theories for a different reason than the others: an apparent absence of any scientific testing (Bailey, 2017). ATA seems to be a collection of supposedly brain-based practices, including learning styles, and movements reminiscent of Brain Gym. This model seeks to provide insight into the training of athletes “to take it to the next level,” by integrating “natural movement” (Action Types, 2013). As is common with such brain-based products, the claims made on behalf of the Action Type Approach are impressive, which might explain why it has been adopted by numerous elite sports groups, including several international football clubs (Action Types, 2013). Unfortunately, no research articles could be found on this method, and requests to the creators and leading advocates resulted in no other sources of research evidence. It is acknowledged that the absence of evidence does not imply evidence of absence, and the lack of peer reviewed study in this case does not mean that the method is not efficacious. However, the apparent lack of published studies raises some doubts about the appropriateness of this strategies' inclusion within evidence-based sports coaching.

### Summary and aim

Other theories included in this study were judged to meet the basic standards necessary for scientific theories. This is not necessarily to suggest that they all embody high levels of correlation and generality. In some cases, such as direct instruction and demonstration, practices embody a substantial amount of empirical support (Hattie, 2008). In others, such as guided discovery and growth mindset, the evidence base is less-strong or context-specific, but still these ideas broadly reflect the criteria for scientific theories discussed above. Table 1 summarizes the ideas and practices included in the survey, and judgments about their scientific credibility, tested against the criteria for pseudoscientific theories suggested above. As such, the aim of this study was to examine British and Irish sports coaches' beliefs and knowledge of pseudoscientific ideas about learning and the brain. Essentially, the ideas examined in this study fall into three broad categories: (1) Scientific (i.e., Direct Instruction Demonstration, Goal-setting, Growth Mindset, and Guided Discovery), (2) Mixed (i.e., Learning Styles and MBTI), (3) Pseudoscience (i.e., ATA, Brain Gym, and NLP). The ideas categorized as “scientific” were each judged to meet *none* of the criteria identified to be associated with pseudoscientific. We believed it was both fair and accurate to differentiate between those ideas that met *all* of the criteria for pseudoscience, and those that met only *some* of them (see Table 1). Numerous commentators on this issue have argued that the distinction between science and pseudoscience is rarely absolute (e.g., Lilienfeld et al., 2012; Monton, 2013), we felt it important to acknowledge this in our analysis. So, while both sets of ideas included in the latter two categories could be considered pseudoscientific, we recognize and maintain a difference in terms of degrees of correspondence to identified criteria.

**Table 1:** Evaluation of brain-based and learning theories against characteristics of pseudoscientific theories (Lilienfeld et al., 2003; Koertge, 2013; Main sources: Bailey, 2017).

	**Unfalsifiab-ility**	**Absence of self-correction**	**Overuse of *ad hoc* immunizing tactics**	**Absence of connectivity**	**Use of obscurantist language**	**Over-reliance on anecdotes and testimonials**	**Evasion of peer review**	**Emphasis on confirmation rather than refutation**
ATA	?	?	?	Y	Y	Y	Y	?
Brain Gym	N	?	Y	Y	Y	Y	Y	Y
Demonstrations	N	N	N	N	N	N	N	N
Direct instruction	N	N	N	N	N	N	N	N
Goal-setting	N	N	N	N	N	N	N	N
Growth Mindset	N	N	N	N	N	N	N	N
Guided discovery	N	N	N	N	N	N	N	N
Learning Styles	N	Y	N	Y	N	Y	N	Y
MBTI	N	N	?	Y	N	Y	Y	Y
NLP	Y	Y	Y	Y	Y	Y	Y	Y

*Y, Evidence of characteristic; N, Little or no evidence of characteristic; ?, Insufficient evidence*.

## Materials and methods

### Participants

The sample comprised 545 coaches from the United Kingdom and Ireland (England, *n* = 345; Ireland, *n* = 112; Northern Ireland, *n* = 41; Scotland, *n* = 31; Wales, *n* = 16). Four-hundred-and thirty-six coaches were male and 109 female. Coaches were categorized as being: 18–24 (*n* = 64), 25–34 (*n* = 120), 35–44 (*n* = 155), 45–54 (*n* = 126), 55–64 (*n* = 54), 65–74 (*n* = 23) or 75 years and older (*n* = 3). The coaches' qualifications were: none (*n* = 27) Level 1 (*n* = 77), Level 2 (*n* = 184), Level 3 (*n* = 152), or Level 4 (*n* = 79). Coaches were employed full-time (*n* = 118), part-time (*n* = 164), volunteers (*n* = 234), or other (*n* = 29). Coaches were involved in coaching in the following sports: soccer (*n* = 138), rugby union (*n* = 67), Gaelic football (*n* = 46), fencing (*n* = 39), swimming (*n* = 26), golf (*n* = 24), cricket (*n* = 20), hockey (*n* = 20), hurling/camogie (*n* = 19), athletics (*n* = 18), netball (*n* = 15), martial arts (*n* = 12), or others (e.g., tennis, rugby league, basketball; *n* = 101).

### Procedure

Sports coaches were approached to participate in the research project via social media. Messages were posted on Twitter and Facebook with a link to the opening page of an online survey tool (http://www.surveymonkey.net). The research was presented as a study of coaches' knowledge and understanding about learning, coaching and the brain. Terms such as “pseudoscience” and “neuromyth” were not mentioned in the information for coaches. Those who chose to participate were invited to agree with a statement of ethics (explaining consent, confidentiality and anonymity), and followed a link to the online survey. Average completion time was 30 min.

### Measures

Participants provided background information about their gender, age, and home country base, as is generally considered standard in surveys of this kind (Gilovich et al., 2006). They were also asked to identify their main sport (as many coaches work in more than 1 sport) their highest level of coaching qualification (coaching qualifications in the UK and Ireland generally begin at Level 1 and go up to Level 4, based on normatively assessed statements of competence), and their employment status as a coach.

#### Interest/awareness of neuroscience

Participants were then asked whether they “come across any of the following ideas or practices in coach education/professional development settings?” Participants responded to list that included 10 different approaches (e.g., goal setting, learning styles, direct instruction, NLP, guided discovery, brain gym, demonstrations, MBTI, growth mindset, and ATA). The compilation of the list of theories began with a survey of empirical studies of evidence-based pedagogical theories (e.g., Hattie, 2008; Carter et al., 2012), and books on educational neuroscience (Della Sala, 2007; Della Sala and Anderson, 2012; Mareschal et al., 2014). The list of statements about the brain and learning was based on a review of studies of prevalence of “neuromyths” and pseudoscience in school education (e.g., Pickering and Howard-Jones, 2007; Dekker et al., 2012; Dündar and Gündüz, 2016; Ferrero et al., 2016; see Table 2 for the list of statements). Obviously irrelevant statements and theories for the present study (e.g., those that with context- or subject-specific aspects of schooling) were removed from the draft lists. Members of a closed Facebook Coaching group (Coaching Science), specialist coaching groups on Twitter and LinkedIn were also invited to list “theories, ideas and practices” that they had “experienced, read or heard about.” This resulted in the addition of one more theory (i.e., Action Types Approach). Working definitions of these theories are given in Appendix 1, along with indicative references consulted. Questions were answered on a 6-Point Likert anchored at 1 “*strongly agree*” and 6 “*strongly disagree*.” Following this, participants were asked to “Please indicate whether or not you use the following ideas or practices in your coaching” The same 10 approaches that were included in the previous question were used. Coaches responded on 5-point Likert-type scale for each of the 10 approaches on a 5-point Likert-Type Scale anchored at 1 “*always use it*” and 5 “*never use it*.”

**Table 2:** Percentage of correct and incorrect responses for each neuromyth and each general assertion about the brain.

	**Correct (C)/Incorrect (I)**	**Agree (%)**	**Disagree (%)**	**Do not know (%)**
**NEUROMYTH**				
Individuals learn better when they receive information in their preferred learning style (e.g., visual, auditory, kinesthetic)	I	62.3	32.2	5.5
Differences in hemispheric dominance (left brain, right brain) can help explain individual differences amongst learners	I	42.5	20.4	37.1
Short bouts of coordination exercises can improve integration of left and right hemispheric brain function	I	50.5	5.0	44.2
Children are less attentive after consuming sugary drinks, and/or snacks	I	54.9	16.9	28.2
There are critical periods in childhood after which certain things can no longer be learned	I	16.6	65.7	17.7
We only use 10% of our brain	I	22.6	43.4	34.0
**GENERAL ASSERTION**				
Vigorous exercise can improve mental function	C	5.7	81.0	13.3
Boys have bigger brains than girls	C	60.0	6.5	33.6
The left and right hemisphere of the brain always work together	C	37.3	18.5	44.3
We use our brains 24 h a day	C	7.8	77.6	14.6
Extended rehearsal of some mental processes can change the shape and structure of some parts of the brain	C	6.1	61.9	32.0
The brains of boys and girls develop at the same rate	I	7.0	60.3	32.7
There are sensitive periods in childhood when it's easier to learn things	C	4.4	81.0	14.6
Learning occurs through modification of the brains' neural connections	C	2.8	65.9	31.4

*N = 545*.

#### Prevalence of neuromyths and knowledge about the brain

The participants were finally presented with a list of 14 statements about learning and the brain (see Table 2). Six of these statements were neuromyths, as defined by OECD (2002), Howard-Jones (2014), and Dekker et al. (2012; e.g., “Individuals learn better when they receive information in their preferred learning style [e.g., auditory, visual, kinesthetic]” and “We only use 10% of our brain”). The other statements were general assertions about the brain (e.g., “Vigorous exercise can improve mental function” and “We use our brains 24 h a day”). The presentation order of myth and knowledge assertions was randomized. Answer options were “incorrect,” “correct,” or “do not know.” We examined the percentage of incorrect answers to neuromyth assertions (where a higher percentage reflects more belief in myths) and the percentage of correct responses to general assertions.

### Data analysis

First, the means and standard deviations for each of the key variables were calculated. *T*-tests investigated gender differences in beliefs in neuromyths and general assertions about the brain. Next, multiple regression analyses quantified the relationships between a series of predictors and the likelihood to believe in neuromyths. The first regression included demographics (gender, age, and coaching qualifications) and percentage of general assertions answered correctly as predictors. The second regression included attitudes toward using information about the brain in relation to coaching as predictors. The third regression included types of learning-based and brain-based Ideas as predictors. Finally, two ANOVAs compared country differences in both neuromyths and general assertions. As this study was exploratory, we did not formulate specific hypotheses.

## Results

### Interest/awareness of neuroscience

Overall, coaches agreed that a better understanding of the brain helped with the following: the planning of sports coaching sessions (*M* = 1.75, *SD* = 0.75; 1 = strongly agree, 5 = strongly disagree); the delivery of sports coaching sessions (i.e., coaching; *M* = 1.62, *SD* = 0.68); the assessment of players'/athletes' learning and development (*M* = 1.50, *SD* = 0.65). Coaches reported receiving information about the role of the brain from a range of sources, including: the media (20.0%), courses delivered by sport organization/national governing body (44.2%), conferences (38.9%), academic journals (54.1%), professional journals (28.8%), books (60.7%), and commercial products or programs (4.6%).

Coaches reported using the following learning-based and brain-based ideas frequently: goal-setting (*M* = 1.95, *SD* = 0.87), Learning Styles (*M* = 2.63, *SD* = 1.44), direct instruction (*M* = 2.23, *SD* = 0.93), guided discovery (*M* = 2.36, *SD* = 1.22), Brain Gym (*M* = 2.41, *SD* = 1.01), demonstrations (*M* = 1.67, *SD* = 0.90), and growth mindset (*M* = 2.86, *SD* = 1.48). The following psychological resources were used infrequently: neuro-linguistic programming (NLP) (*M* = 4.12, *SD* = 1.16), Myers-Briggs Type Inventory (MBTI) (*M* = 4.52, *SD* = 0.89), action types approach (ATA) (*M* = 4.25, *SD* = 1.17). Coaches' exposure to the Learning-based and Brain-based Ideas is presented in Table 3 and includes coaches' overall exposure, as well as specific sources of this exposure.

**Table 3:** Coaches exposure to different learning-based and brain-based ideas.

	**Percentage that have come across these ideas/practices**	**Core coaching qualification courses delivered by your sports organization**	**Other courses run by your sports organization**	**Conferences run by your sports organization**	**Coaching courses delivered by other organizations**	**Other courses delivered by other organizations**	**Other conferences**
Goal setting	91.9	57.7	26.1	25.7	32.5	33.5	17.0
Learning styles	88.8	56.8	21.3	18.6	25.8	33.7	14.5
Direct instruction	82.0	73.8	25.5	19.9	20.6	25.7	11.6
NLP	53.9	14.6	14.6	9.9	18.7	47.3	33.7
Guided	74.9	52.2	25.2	19.6	24.3	33.8	19.6
Brain gym	35.4	13.5	9.8	8.3	14.0	43.0	40.4
Demonstrations	87.9	82.3	33.6	24.4	28.6	28.4	15.2
MBTI	44.6	17.3	9.5	9.1	20.6	46.5	32.5
Growth	63.3	36.8	25.2	21.4	29.0	43.5	38.8
ATA	35.2	26.0	14.6	14.6	20.3	40.1	28.1

*All values are percentages (%)*.

### Prevalence of neuromyths

Table 2 presents the percentage of responses of agreement and disagreement to each neuromyth. Overall, coaches agreed with 41.6% (*SD* = 26.3%) of the statements promoting myths. The most prevalent neuromyth was “Individuals learn better when they receive information in their preferred learning style (e.g., auditory, visual, or kinesthetic).” 62% of coaches believed this neuromyth. The most successfully identified neuromyth was “There are critical periods in childhood after which certain things can no longer be learned,” which 65.7% of coaches identified. Women on average (9.74%) agreed with more neuromyths than men [*t*_(533)_ = 3.45, *p* < 0.01].

The results of the multiple regression analyses are reported in Tables 4–6. These analyses tested the variables that were associated coaches believing in neuromyths (i.e., answering incorrectly). Female coaches were more likely to agree with neuromyths than male coaches. Coaches with a better knowledge of the brain were more likely to believe neuromyths. The coaches who agreed that understanding the brain could help with “The assessment of players'/athletes' learning and development” were also more likely to believe in neuromyths, as were coaches who frequently used the questionable techniques discussed earlier. Those coaches who used learning styles less frequently and used guided discovery more frequently were more likely to believe the neuromyths.

**Table 4:** Multiple regression predicting neuromyths from demographics and general assertions.

	**Neuromyths**	
	***R*^2^**	**β**
	0.08^***^	
Gender		−0.14^**^
Age		0.04
Qualifications		0.09
% of assertions correct		0.22^***^

*N = 520. Female = 1, Male = 2*,

** *p < 0.01*.

*** *p < 0.001*.

**Table 5:** Multiple regression predicting neuromyths from attitudes to situations that would be improved by an understanding of the brain.

	**Neuromyths**	
	***R*^2^**	**β**
	0.03^**^	
The planning of sports coaching sessions		−0.03
The delivery of sports coaching sessions (i.e., coaching)		−0.11
The assessment of players'/athletes' learning and development		0.20^**^

*N = 519. Betas were reversed to enhance interpretation (i.e., a positive beta signifies a positive relationship)*.

** *p < 0.01*.

**Table 6:** Multiple regression predicting neuromyths from use of learning-based and brain-based ideas.

	**Neuromyths**	
	***R*^2^**	**β**
	0.27^***^	
Goal-setting		−0.07
Learning Styles		−0.45^***^
Direct instruction		0.03
Neuro-linguistic Programming (NLP)		−0.01
Guided discovery		0.11^*^
Brain Gym		−0.10
Demonstrations		−0.05
Myers-Briggs Type Inventory (MBTI)		−0.01
Growth Mindset		0.06
Action Types Approach (ATA)		−0.01

*N = 515. Betas were reversed to enhance interpretation (i.e., a positive beta signifies a positive relationship)*.

* *p < 0.05*.

*** *p < 0.001*.

### Knowledge about the brain

Coaches answered 56.6% (*SD* = 19.9%) of the questions pertaining to general assertions about the brain correctly. Table 2 shows percentages of correct and incorrect responses for each question. The assertions most correctly identified were “Vigorous exercise can improve mental function” and “There are sensitive periods in childhood when it's easier to learn things,” correctly answered by 81.0% of coaches. Whereas the assertion least correctly identified was “Boys have bigger brains than girls,” incorrectly answered by 60.0% of coaches. Moreover, no gender differences were found for general assertions [*t*_(525)_ = 0.64, *p* = 0.52].

### Differences between countries

An ANOVA comparing differences in the percentage of neuromyths answered incorrectly between countries was significant [*F*_(5, 530)_ = 4.33, *p* < 0.01]. However, once the alpha level had been adjusted based on a Bonferroni correction (*p* < 0.005; which was applied because of unequal group sizes and multiple comparisons), no significant differences were found. Furthermore, no country differences in general assumptions were found [*F*_(4, 522)_ = 0.20, *p* = 0.94].

## Discussion

This study is the first to examine the prevalence of pseudoscientific ideas among British and Irish sports coaches. We explored how evidence-based and non-evidence-based ideas regarding learning and the brain were understood by these coaches to enhance their practice. We also explored their exposure to these different theories, and the knowledge of basic neuroscientific information that might be relevant to their work. Findings showed there is a strong support for the introduction of brain-based information into sports coaching and coach education. However, data reveal a relatively high prevalence of “neuromyths” (41.6%). This figure is lower than had previously been found in studies with school teachers (Pickering and Howard-Jones, 2007; Dekker et al., 2012). Nevertheless, the figure is substantial enough to warrant concern, because it is likely that these beliefs will shape coaching philosophy and practice. As with these earlier studies, the most commonly held pseudoscientific belief was that “Individuals learn better when they receive information in their preferred learning style (e.g., auditory, visual, or kinesthetic),” which was held by 62% of the sample.

The reason for the prevalence of questionable ideas among coaches and teachers is not clear. The coaches in our study came from a much more diverse range of educational backgrounds than the teachers who took part in earlier research, and it could be that teachers' graduate education expose them to ideas about the brain, but without sufficient depth to immunize them against neuromyths. The finding that there is a correlation between general knowledge about the brain and susceptibility to neuromyths among trainee teachers (Dekker et al., 2012), and that people with some neuroscientific knowledge (e.g., who took an introductory cognitive neuroscience course work) are vulnerable to questionable neuroscientific explanations as the general public (Macdonald et al., 2017), offer some support for this hypothesis. Coach education is much less centrally regulated than teacher education in the UK and Ireland (Duffy et al., 2013; North et al., 2016), and evidence presented in this study shows that coaches rely upon a wide range of information sources, including books, conferences, journals, the popular press, and social networking sites. It might be the case that the laissez-faire approach to professional development described by the coaches in this study means that they are not well-placed to make judgments regarding the scientific quality of the sources of information they access. Experimental research has shown that some people are especially vulnerable to the sort of strategies employed by advocates of pseudoscience described in this paper (Pennycook et al., 2015), and that people are more likely to accept research findings when they are accompanied by attractive brain images and brain-based explanations, even when these are incorrect or irrelevant (McCabe and Castel, 2008; Weisberg et al., 2008). Collins and Bailey (2013) claimed that sports organizations are particularly vulnerable to “scienciness,” or “the illusion of scientific credibility and validity that provides a degree of authority to otherwise dubious ideas” (p. 2). In the context of research into learning and the brain, this suggests that people with little or no neuroscientific education will be inclined to make misjudgments of presented evidence, and will find it difficult to recognize misconceptions about brain research (Dekker et al., 2012). The popular media has been identified in previous research as a source of over-simplified or over-interpreted representations of the brain (OECD, 2002; Beck, 2010), and while this was not identified by the coaches as an especially significant influence, it is likely that they were as vulnerable to widely promulgated misconceptions as the rest of the population.

Within the context of coach education, significant directions of transfer are likely to be from experienced to inexperienced practitioners, and coach educators to trainee coaches, and through teaching materials endorsed by national governing bodies for sport (Stoszkowski and Collins, 2016). Social media and online blogs have also been identified as popular repositories of information (Stoszkowski et al., 2017). Pseudoscientific beliefs are likely to find hospitable environments in many of these contexts, as they share many of the characteristics that have been found to characterize “mimetically fit” cultural units (von Bülow, 2013). Findings from business psychology and social physics suggested that, all other things being equal, ideas will be fitter and spread more effectively if: they are attention-grabbing or surprising; they appealed to personal interests; they are relatively simple; they propose a simple X–Y causality; they provoke action (Heath and Heath, 2008; Pentland, 2014). These characteristics seem to capture many of the pseudoscientific beliefs surveyed in this study. For example, the myth that people typically use just 10% of their brains holds within it the promise of extra ordinary improvements in cognitive functioning and physical performance (once the necessary triggering mechanism has been found). A similar appeal might attract people to programs such as Brain Gym, which promises substantial improvement to performance in a range of domains (Dennison and Dennison, 1994).

The notion that everyone has a dominant or preferred learning style was found to be the most commonly held non-evidence-based theory in this study, as it was in earlier surveys with teachers (e.g., Dekker et al., 2012; Macdonald et al., 2017). Perhaps this is because the idea relates to all four of the characteristics of easily spread beliefs: it is based on a surprising, but not entirely implausible model of brain organization; it is psychologically attractive, both in terms of its claimed benefits and because it resonates with the attribution of difficulties with learning two variables largely outside of the individuals control (such as inappropriate pedagogy); it offers a simple and memorable framework; and it presents an intuitively appealing course of action (adaptive learning and teaching strategies to reflect the supposed preferred modalities). In addition, they are explicitly brain-based and “sciency.”

The proliferation of pseudoscientific beliefs among countries is cause for concern, as many of the ideas discussed in this study directly relate to coach and participant learning. Misconceptions about learning and the brain could, therefore, have a harmful effect on participant outcomes. Pashler et al. (2009) argued that teaching according to identified learning styles might not just be theoretically ill-advised, but could be deleterious for learning, because learners are guided away from non-preferred modalities that are likely to facilitate greater cognitive load. In addition, some pseudoscientific beliefs are packaged as commercial programs, and the adoption directs time and funding away from empirically supported alternatives (Carter et al., 2011).

One way of addressing this issue is through education. The Organization for Economic Cooperation, and Development (OECD, 2002) was one of the first agencies to draw attention to the prevalence and potentially harmful influence of neuromyths, and made a case for the inclusion of “brain research in education and other contexts” (p. 258). This is a sentiment endorsed by several commentators on this topic, whilst also stressing the need for bidirectional collaborations between scientists and professional groups (Ansari and Coch, 2006; Coltheart and McArthur, 2012; Howard-Jones, 2014). However, enthusiasm for greater education as a solution to the prevalence of pseudoscience is tempered by the finding that teachers with more general knowledge about the brain can become more likely to believe questionable ideas (Macdonald et al., 2017). So, further research into effective educational practices in this area it is vital. Insofar as coach education is likely to be a part of the solution to the problem of pseudoscientific beliefs and practices, there is a need to enhance professional development and inter-disciplinary scientist-practitioner partnerships to reduce miscommunications in the future. The finding that coaches are eager to extend their understanding of applied neuroscience is encouraging, and suggests that they would be willing to engage with genuine science, if presented in an accessible manner. Therefore, the complex and challenging integration of neuroscience in sports coaching is most likely to follow genuine collaborations between practitioners and scientists. More generally, coach education would be strengthened by encouraging the cultivation of a healthy skepticism, which the popular science writer, Sagan (1987) described as “an exquisite balance between two conflicting needs: the most skeptical scrutiny of all hypotheses that are served up to us and at the same time a great openness to new ideas.” One method for doing this is to explicitly discuss the distinction between science and pseudoscience (Lilienfeld et al., 2012), perhaps using some of the more clear-cut examples of the latter as case studies. As the first of its kind with sports coaches, this study can be understood as a contribution to these endeavors.

A complementary approach is to address the organizations that promote and implicitly endorse non-evidence-based practices. From the perspective of the sports coaches in this study, the most influential bodies are the National Governing Bodies (NGB) that lead individual sports, and usually regulate accreditation and training. This is a more intractable challenge, and whilst individuals within their organizations could foster change, large-scale improvement would take a considerable time, and might ultimately prove unsuccessful. Based on the fact that almost every major NGB in the UK and Ireland receive funds from central government, and that coaches are required to attend NGB programs, it might be possible to make the implementation of evidence-based practices a condition of this funding. Aside from the likely improvement to coaching performance (and, indirectly, sporting success), this step could be justified on ethical grounds as forcing people to undertake courses that include pseudoscience is morally indefensible. A less draconian alternative would be to introduce an advisory body for NGBs, somewhat similar to the UK's National Institute for Health and Care Excellence (NICE), which could offer evidence-based guidance on sports coaching (as well as other aspects of sport participation and performance). Precisely this proposal was made to the UK's All Party Commission on Physical Activity (2014) by the first author of this article, and was accepted by the Commission as a formal recommendation. Sadly, the Commission's Final Report was rejected by the national Government of the day.

### Limitations and future directions

The present study has several limitations. First, data were gathered from British and Irish sports coaches, and the current state of coach education is sufficiently varied to mean that international generalizations are unwise. For example, compulsory coach education and accreditation is currently far from universal, both in terms of statutory provision and content (North et al., 2016). Consequently, further system-specific and comparative studies are required for a more complete picture. Second, the survey was administered online. This is now a common practice (Biffignandi and Bethlehem, 2012). Nevertheless, the recruitment strategy focused on countries who accessed specialist social sports media sites. Anecdotal evidence suggests that some participants were directed to the survey by organizations, and others from posts to sports coaching groups. Hence, the sample was probably over-selected for individuals with existing interests in professional development other groups may have been over-represented in the sample. Third, an additional potential difficulty with online surveys is that it is impossible to rule out the possibility that respondents carry out research to help them answer certain questions. This issue is especially relevant to the data reported in Table 2, and consequently, these answers out to be accepted with caution. This is not a limitation restricted to online surveys: any non-supervised data-gathering tool suffers from the same concern. Fourth, the survey results do not make it clear when coaches were exposed to the different learning-based and brain-based ideas, and it could have been many years ago, and possibly before developments in coach education. Future research with more representative samples of coaches and with sub-populations (e.g., novice coaches, coach educators), and qualitative data analysis, aim to address some of the limitations of the present study. Finally, our sampling procedure may have impacted upon the results. We used a convenience sampling methodology via social media. As such, coaches who have an interest or knowledge of the concepts we assessed in the present study may have been more inclined to participate. Given this was the first study to assess these variables among coaches, we felt it was an entirely appropriate sampling procedure. In the future, however, scholars could adopt a randomized method.

In summary, this is the first study of the prevalence of pseudoscientific beliefs amongst sports coaches. The study provides a useful baseline for subsequent empirical studies of their prevalence, content and dissemination, and insight into the uptake of these beliefs in a relatively new field of study. Findings show that sports coaches, like school teachers, can find it difficult to distinguish between pseudoscience from genuine scientific research. Questionable ideas and practices, like learning styles, neurolinguistics programming, and Myers-Briggs are not simply acquired by coaches through their own personal interest, they are often actively promoted by sports organizations. So, this situation requires changes at the level of both the content of coach education programs, which ought to have secure evidence base, and context of the national governing bodies, in which pseudoscience is allowed to thrive.

## Ethics statement

This study was carried out in accordance with the recommendations of the British Psychological Society with written informed consent from all subjects. All subjects gave written informed consent in accordance with the Declaration of Helsinki. The protocol was approved by the School of Life Sciences, University of Hulls ethics committee.

## Author contributions

RB conceptualized, collected data, and contributed towards writing the paper, DM analyzed and contributed to writing, EC contributed to writing, and AN contributed to data collection and writing.

### Conflict of interest statement

The authors declare that the research was conducted in the absence of any commercial or financial relationships that could be construed as a potential conflict of interest. The reviewer, SM, and handling Editor declared their shared affiliation.
