# Supplementary material for: The Prevalence of Pseudoscientific Ideas and Neuromyths Among Sports Coaches
Source: Front Psychol. 2018 May 2;9:641. doi: 10.3389/fpsyg.2018.00641 (PMC5941987; doi:10.3389/fpsyg.2018.00641)
Supplement: Supplementary file 1 [file Table_1.docx]

APPENDIX 1: Evidence-based (E) and questionable (Q) ideas - indicative references

| Action Types Approach (ATA) (Q) | A collection of supposedly brain-based practices; seeks to provide insight into the training of athletes by integrating natural movement | No empirical studies found. |
| --- | --- | --- |
| Brain Gym (Q) | A popular commercial brain-based program founded on the premise that learning problems are caused when different sections of the brain and body do not work in a coordinated manner, thereby blocking a student’s ability to learn. | Hyatt (2007); Ruhaak and Cook (2016) |
| Demonstrations (E) | A form of teaching/coaching for behavior change through processes such as observational learning, imitation and emulation. | D’Innocenzo et al. (2016); Evans and Fitzgerald (2017) |
| Direct instruction (E) | Instruction that is teacher directed, is goal oriented, and requires deliberate implementation | Hattie (2008); McMullen and Madelaine (2014) |
| Goal-setting (E) | The process of identifying an objective to accomplish and establishing measurable goals and timeframes towards it | Díaz-Ocejo and Mora-Mérida (2013); Locke and Latham (2015) |
| Growth Mindset (E) | A theory proposed by psychologist Carol Dweck as a way to understand the effects of the beliefs that individuals hold about the nature of ability. | Claro et al. (2016); Dweck, (2000) |
| Guided discovery (E) | A teaching strategy in which students take some of the responsibility for their own learning and inquiry, whilst supported by the teacher/coach | Janssen et al. (2014); Lazonder and Harmsen, 2016) |
| Learning Styles (N) | The general claim that people learn in different qualitatively ways, and that formal experiences can be tailored to the individual learning style of the student | Coffield et al. (2004); Rohrer and Pashler (2012) |
| Myers-Briggs Type Inventory (MBTI) (Q) | A self-report questionnaire claiming to indicate preferences in how people perceive the world around them and make decisions | Barbuto (1997); Paul (2010) |
| Neuro-linguistic Programming (NLP) (Q) | Related approaches to communication, personal development, and behavior change. | Beyerstein (1990); Witkowski (2010) |
